# Immediate Blood Draw for CD4+ Cell Count Is Associated with Linkage to Care in Durban, South Africa: Findings from *Pathways to Engagement in HIV Care*

**DOI:** 10.1371/journal.pone.0162085

**Published:** 2016-10-05

**Authors:** Susie Hoffman, Theresa M. Exner, Naomi Lince-Deroche, Cheng-Shiun Leu, Jessica L. Phillip, Elizabeth A. Kelvin, Anisha D. Gandhi, Bruce Levin, Dinesh Singh, Joanne E. Mantell, Kelly Blanchard, Gita Ramjee

**Affiliations:** 1 HIV Center for Clinical and Behavioral Studies, New York State Psychiatric Institute, Columbia University, New York, New York, United States of America; 2 Department of Epidemiology, Mailman School of Public Health, Columbia University, New York, New York, United States of America; 3 Ibis Reproductive Health, Johannesburg, South Africa; 4 Department of Biostatistics, Mailman School of Public Health, Columbia University, New York, New York, United States of America; 5 South African Medical Research Council, HIV Prevention Research Unit, Durban, South Africa; 6 Epidemiology & Biostatistics Program, CUNY Graduate School of Public Health and Health Policy, City University of New York, New York, New York, United States of America; 7 Ibis Reproductive Health, Cambridge, Massachusetts, United States of America; Katholieke Universiteit Leuven Rega Institute for Medical Research, BELGIUM

## Abstract

**Background:**

Timely linkage to care by newly-diagnosed HIV+ individuals remains a significant challenge to achieving UNAIDS 90-90-90 goals. Current World Health Organization (WHO) guidelines recommend initiating anti-retroviral treatment (ART) regardless of CD4+ count, with priority given to those with CD4+ <350 cells/μl. We evaluated the impact of not having a day-of-diagnosis CD4+ count blood draw, as recommended by South African guidelines, on time to linkage, using data from a prospective cohort study.

**Methods:**

Individuals (N = 2773) were interviewed prior to HIV counseling and testing at three public sector primary care clinics in the greater Durban area; 785 were newly-diagnosed and eligible for the cohort study; 459 (58.5%) joined and were followed for eight months with three structured assessments. Linkage to care, defined as returning to clinic for CD4+ count results, and day-of-diagnosis blood draw were self-reported.

**Results:**

Overall, 72.5% did not have a day-of-diagnosis CD4+ count blood draw, and 19.2% of these never returned. Compared with a day-of-diagnosis blood draw, the adjusted hazard ratio of linkage (AHR_linkage_) associated with not having day-of-diagnosis blood draw was 0.66 (95%CI: 0.51, 0.85). By 4 months, 54.8% of those without day-of-diagnosis blood draw vs. 75.2% with one were linked to care (chi-squared *p* = 0.004). Of those who deferred blood draw, 48.3% cited clinic-related and 51.7% cited personal reasons. AHR_linkage_ was 0.60 (95%CI: 0.44, 0.82) for clinic-related and 0.53 (95%CI: 0.38, 0.75) for personal reasons relative to having day-of-diagnosis blood draw.

**Conclusions:**

Newly-diagnosed HIV+ individuals who did not undergo CD4+ count blood draw on the day they were diagnosed—regardless of the reason for deferring—had delayed linkage to care relative to those with same-day blood draw. To enhance prompt linkage to care even when test and treat protocols are implemented, all diagnostic testing required before ART initiation should be performed on the same day as HIV testing/diagnosis. This may require modifying clinic procedures to enable overnight blood storage if same-day draws cannot be performed, and providing additional counseling to encourage newly-diagnosed individuals to complete day-of-diagnosis testing. Tracking HIV+ individuals via clinic registries should commence immediately from diagnosis to reduce these early losses to care.

## Introduction

In most low and middle income countries (LMIC) current practice is to stage individuals’ HIV disease, either clinically or through CD4+ count, as a first step in care. In September 2015, World Health Organization (WHO) guidelines were revised to recommend that all HIV+ individuals be placed immediately on antiretroviral therapy (ART) regardless of CD4+ cell count, and that those with CD4+ count less than 350 cells/μl be prioritized for ART initiation [1]. This guidance draws from the findings of the START [2] and TEMPRANO [3] studies, both of which demonstrated a substantial benefit of ART on HIV-related morbidity regardless of the stage of disease at which treatment was initiated. Regardless of the new WHO guidelines, baseline CD4+ count testing will likely continue in many LMIC, both (1) to prioritize individuals for ART initiation when resources are limited, and (2) to determine need for opportunistic infection prophylaxis, especially given that large proportions of individuals are diagnosed and/or enrolled in care with very low CD4+ counts [4–6]. Earlier studies noted low completion rates of this step [7–10], and one demonstrated that HIV+ individuals who were not staged by WHO clinical guidelines on the same day as their HIV diagnosis were less likely to complete ART eligibility assessment [10]. Point-of-care CD4+ count testing with provision of the test results at the time of diagnosis has been shown to facilitate more rapid linkage to HIV care [11–13] but remains beyond the capacity of many public sector clinics in LMIC.

During the first decade of ART roll-out in South Africa, treatment thresholds tended to lag behind those recommended by WHO, but in recent years, guidelines generally have been in line with those recommended by WHO [14,15]. During 2010–2013, the period relevant for this study, South Africa moved from a treatment initiation threshold of less than 350 cells/μl only for pregnant women and TB-co-infected adults to this threshold for all HIV+ adults. Notably, beginning in 2010, South African guidelines encouraged blood to be drawn for a CD4+ count assay at the same clinic visit as the initial HIV diagnosis to encourage timely determination of eligibility for ART [16]. The degree to which this policy was implemented, and its effect on time until individuals are linked to care, are unknown.

In a prospective cohort study, ‘Pathways to Engagement in HIV Care for Newly-Diagnosed South Africans’ (hereafter, Pathways to Care), we examined what proportion of individuals had blood drawn for CD4+ count determination on the day of diagnosis, reasons for not doing so on that day, and the relationship between not having a day-of-diagnosis blood draw and time to linkage to care. The results of this analysis provide critical insights into how procedures required for access to care and treatment might promote or delay linkage to those services.

## Methods

The Pathways to Care cohort consisted of newly-diagnosed HIV+ women and men recruited from three public sector primary healthcare clinics (PHC) located in greater Durban, including two sites in urban areas and one in a rural area. KwaZulu-Natal, the province in which Durban is situated, has the highest HIV prevalence in South Africa, with 17.4% (15.8–19.2) of the population aged two years and older infected in 2012 [17]. All three clinics routinely provided a range of primary healthcare services and ART treatment in addition to HIV counseling and testing (HCT). Participants were recruited between November 2010 and May 2012, and were followed for up to eight months after diagnosis to identify factors associated with time to linkage to care.

### Participants

Eligibility criteria included being aged 18 years or older, testing HIV+ on the day of recruitment, not previously having been diagnosed HIV+, and willing to have HIV test results shared with the study interviewer. We excluded individuals who did not anticipate residing in their community for at least one year (to ensure high retention and data quality), were pregnant, did not speak and understand English or isiZulu, or were observed by study staff to be cognitively impaired. Pregnant women and those referred from antenatal care were excluded because they represent a different population of newly-diagnosed individuals; their patterns of diagnosis, referral, and care differ from those of men and non-pregnant women, and studying them would have required a sufficient sample to make inferences around that group alone.

**Fig 1:**
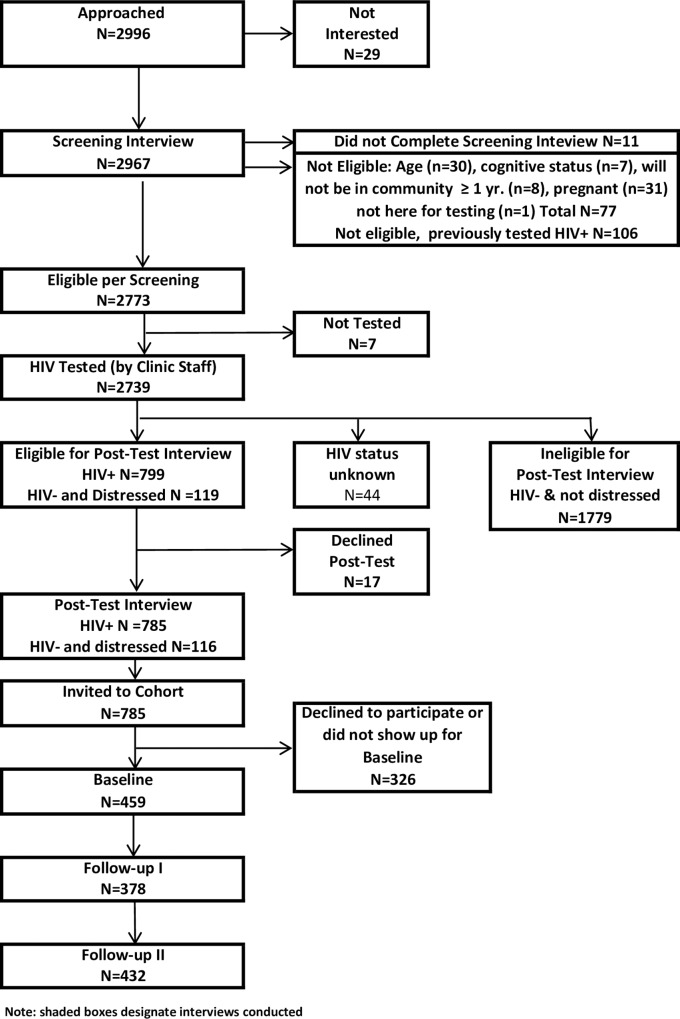
Participant flow for Pathways to Engagement in HIV Care for Newly-Diagnosed South Africans.

To recruit the cohort, all persons presenting to one of the three clinic sites for HIV testing (or every second person if it was not possible to approach all on a given day) were invited to a 20-minute screening interview prior to testing (Fig 1). The screening interview ascertained study eligibility and potential psychosocial determinants of linkage to HIV care. After clinic personnel conducted HCT, individuals who were HIV+ and/or had elevated symptoms of psychological distress on the screening interview as determined by the Kessler-10 [18] were invited to a 5-minute post-test interview, which ascertained perceived barriers to care and interest in ancillary service/support referrals. Distressed individuals were included in the post-test interview so that the status of HIV+ individuals would not be revealed on account of their participation. After the post-test interview, HIV+ individuals were invited to join the cohort study. Participants were reimbursed R40 and R15 (the approximate equivalent of US $5 and $2 at the time) for the screening and post-test interviews, respectively.

Written informed consent was obtained prior to the screening interview; an additional written informed consent was obtained prior to the baseline interview for the cohort study. All procedures were approved by the Institutional Review Board of the NYS Psychiatric Institute/Columbia University Department of Psychiatry and the Biomedical Research Ethics Committee of the University of KwaZulu-Natal.

### Assessment and cohort follow-up

Cohort study participants underwent a structured interview at baseline, which was conducted within 30 days of initial HIV diagnosis, and at four and eight months after diagnosis. Follow-up for the 8-month interview continued until the close of the study to enable maximum retention. Participants who failed to attend interview appointments and who could not be reached by telephone were visited at home by field staff if permission had been granted in advance.

Assessments were conducted in isiZulu by trained interviewers at a designated, private location at or near the clinic where the participant was diagnosed. Referrals for ancillary/supportive services were offered after each interview, and participants were reimbursed R70, R90, and R120 (the equivalent at the time of US $10, $12, and $15) for the baseline, 4-month, and 8-month interviews, respectively.

### Measures

Except for global positioning system (GPS) distance calculations (see below), all cohort data were obtained by self-report, as medical records in these public primary care clinics were not created until individuals initiated ART.

#### Outcome

Linkage to care was defined as returning to the clinic to obtain CD4+ count test results, a linkage indicator that has been used in other investigations [19,20]. Participants were asked at each assessment if they had any non-hospitalization medical visits since the previous interview, and the date, location, and reason for the visit. Based on the self-reported reasons for visits, the interviewer determined whether it appeared that the participant had or had not received his/her CD4+ count test results, and subsequently confirmed this by asking directly. Time to linkage to care was calculated as the number of days from diagnosis to receiving CD4+ count test results, and was converted to fractional weeks for ease of interpretation. Additionally, two dichotomous outcome variables were created: (1) linkage to care by four months and (2) linkage to care by eight months after diagnosis.

#### Predictor–No blood draw for CD4+ count on day of diagnosis

Not having blood drawn for CD4+ count on the same day as receiving one’s HIV-positive test result was the predictor of interest. At the baseline interview, we asked participants if they had a blood draw for CD4+ count on the day of diagnosis, or if not, if they had the blood draw since then, and the date and location of that visit. These questions were repeated at follow-up interviews for those who had not previously reported a blood draw for CD4+ count.

#### Reasons for no CD4+ count blood draw

Individuals who had not had blood drawn for CD4+ count by their baseline interview were asked why they had not had this test. Open-ended responses were assigned to a priori codes, with additional codes added as needed. Codes were grouped into clinic-related and personal reasons.

#### Potential confounders

These included age in years, gender, relationship status, highest year of schooling, employment status, food insecurity, source of income, number of children living at home, travel time to clinic, prior HIV testing, suspected being HIV+, and number of HIV-related symptoms, all measured prior to diagnosis. Additionally, we evaluated as potential confounders the following psychosocial and social cognitive measures, also measured prior to diagnosis: symptoms of psychological distress [18], attitudes toward returning to the clinic to receive CD4+ results [21] (Cronbach’s alpha = 0.879), and HIV-related stigma [22] (Cronbach’s alpha = 0.748). Barriers to care were ascertained after diagnosis; for gender-related barriers we took the mean of three items (‘your partner does not want you to go to the clinic’; ‘you have to take care of children or other family members’; ‘the times the clinic is open are not convenient for you’*)* (Cronbach’s alpha = 0.767).

#### GPS distance measures

At the baseline interview participants identified a landmark near their home (e.g., school, church, local shop) and estimated walking time from home to landmark (<10 minutes, 10–20 minutes, 21–30 minutes, >30 minutes), which we converted to walking distances of 0.42km, 1.25km, 2.08km, 2.92km, respectively. In order to estimate travel distance between home and clinic, we used GPS software to calculate the straight-line and by-roads distance from the landmark to the clinic where diagnosed, and added the estimated walking distance from home to the landmark. If the participant reported having his/her blood drawn for CD4+ count at a clinic other than where they were diagnosed, distance to that clinic was calculated.

### Statistical analyses

We compared eligible individuals who joined the cohort study versus those who did not join using chi-squared, Student’s t-tests, or Mann-Whitney U-tests, as appropriate. Among cohort study participants, we compared those with and without a blood draw at diagnosis with respect to the variables described above, except for GPS distance, which was not assessed at screening.

Kaplan-Meier curves for time to linkage were plotted, and log-rank tests were used to compare the cumulative probability of linkage for those with and without a day-of-diagnosis CD4+ count blood draw, and those with clinic-related and personal reasons for no day-of-diagnosis blood draw relative to day-of-diagnosis blood draw. Individuals were censored at their last interview if they were lost to follow-up, died, or declined to continue with the study.

The primary analysis used Cox proportional hazards models stratified by clinic of recruitment to estimate the log hazard ratio (HR) of linkage to care associated with not having CD4+ count blood drawn on the day of diagnosis and to adjust for confounding variables. We included in adjusted models variables that singly or in combination attenuated the crude estimate by 10% or greater in order to estimate the smallest possible effect of day-of-diagnosis blood draw. In separate models we replaced the dichotomous predictor, blood draw at diagnosis (no/yes), with the 3-category predictor, reason for no blood draw at diagnosis (clinic-related, personal, had day-of-diagnosis CD4+ count blood draw). The 3-category predictor models excluded the observations from 89 individuals who were not asked the reason for no blood draw because they had blood drawn for CD4+ count by the time of their baseline interview, and two who had missing data. To assess if the effect of day-of-diagnosis blood draw differed according to gender, we examined Cox models stratified by gender and tested the significance of an interaction term between gender and day-of-diagnosis blood draw.

For the categorical outcomes (linked by four months, linked by eight months), we used Mantel-Haenszel chi-squared statistics and conditional logistic regression, conditioning on clinic of recruitment. Participants were included in these analyses if they had an 8-month follow-up interview or, if missing the 8-month follow-up interview, it was possible to ascertain from baseline or 4-month interview data that they had a blood draw for CD4+ count by eight months.

## Results

### Participant recruitment and retention

Between November 2010, and May 2012, 2967 individuals who presented for HCT at one of the three study clinics agreed to be screened for study eligibility (Fig 1). Of these, 183 were ineligible (mostly because they had been diagnosed previously) and 11 did not complete the screening interview. Of the remaining 2773, 785 (28.2%) tested HIV+, completed the post-test interview, and were invited to participate in the cohort study. Of those invited, 459 (58.5%) enrolled in the study. Between the 459 eligible participants who joined the cohort study and the 326 who were eligible and did not join, the only statistically significant differences were by clinic of recruitment (the rural clinic had higher acceptance) and relationship status (those living with a spouse or partner had lower acceptance) (S1 Table).

Four-month interviews were completed by 378 (82%) and 8-month interviews were completed by 432 (94.1%) of those enrolled. Overall, 443 (96.5%) had at least one follow-up interview, with a median of 39.7 person-weeks of follow-up in the entire cohort. The outcome was able to be determined in 439 of 459 individuals (95.6%). Eight individuals lost to follow-up after the baseline interview were determined to have died.

### Description of the population and blood draw for CD4+ count at diagnosis

The socio-demographic characteristics of the cohort study participants overall and by day-of-diagnosis blood draw are shown in Table 1. The median age was 29 years (IQR: 25–35), and 67% were women. The largest proportion (65%) was not cohabiting but married or in a relationship. Participants faced a number of socioeconomic challenges: one-quarter (25%) had completed high school, less than one-third (31%) were employed, and 46% reported food insecurity. Sixty-one percent had not been tested previously for HIV.

**Table 1:** Description of the population at baseline, overall and by whether blood was drawn for CD4+ count on the day of diagnosis, 459 newly-diagnosed HIV+ women and men, Durban, South Africa, 2010–2012.

	ALL	Blood Draw at Diagnosis	No Blood Draw at Diagnosis	p-value^1^
	N = 459	N = 126	N = 333	
**DEMOGRAPHIC AND SOCIOECONOMIC CHARACTERISTICS**				
**Gender, %**				
Women	67.3	61.9	69.4	0.159
Men	32.7	38.1	30.6	
**Age in years, median [Interquartile Range (IQR)]**	29 [25–35]	30 [27–37]	29 [25–34]	0.043
**Age group, %**				
< = 24	21.6	14.3	24.3	0.033
25 to 28	21.4	27.0	19.2	
29 to 31	18.1	15.1	19.2	
32 to 36	19.2	18.3	19.5	
> = 37	19.8	25.4	17.7	
**Relationship status,%**				
Not married/in a relationship	11.1	14.3	9.9	0.230
Married/in a relationship—not living together	64.5	58.7	66.7	
Married/in a relationship—living together	24.4	27.0	23.4	
**Number of children living with you, median [IQR]**	1 [0–2]	1 [0–2]	1 [0–2]	0.229
**Highest year of school,%**				
8th grade or less	25.1	23.8	25.5	0.569
9th-11th grade	48.6	52.4	47.1	
Matriculated (completed h.s.)	16.1	12.7	17.4	
More than high school	10.2	11.1	9.9	
**Employment, %**				
Employed full/part time or self-employed	30.5	38.9	27.3	0.022
Unemployed, unable to work, student	69.5	61.1	72.7	
**Food insecurity, %**				
Never	53.6	50.0	55.0	0.063
Seldom	10.5	16.7	8.1	
Sometimes	23.1	22.2	23.4	
Often	12.9	11.1	13.5	
**Income Source,%**				
Has income source or government grant	96.3	100.0	94.9	0.021
No income source & no government grant	3.7	0.0	5.1	
**Travel time to clinic, %**				
<1/2 hour	45.5	38.9	48.0	0.098
≥1/2 hour	54.5	61.1	52.0	
**Distance to clinic by roads km., median [IQR]**	3.1 [1.7–6.9]	3.8 [1.7–11.1]	3.0 [1.7–6.0]	0.043
**TESTING CHARACTERISTICS**				
**Ever tested (negative) before this test, %**				
Yes	38.6	43.7	36.7	0.212
No	61.4	56.3	63.3	
**Suspected HIV-positive, %**				
Yes/unsure	61.0	67.5	58.6	0.101
No	39.0	32.5	41.4	
**Number of HIV-related symptoms, %**				
None	53.2	50.0	54.4	0.530
One	20.5	23.8	19.2	
More than one	26.4	26.2	26.4	
**PSYCHOSOCIAL and COGNITIVE FACTORS**				
**Symptoms of psychological distress, %**				
Not elevated (<16)	87.7	85.5	88.5	0.473
Elevated (≥ 16)	12.3	14.5	11.5	
**HIV-related stigma, median [IQR]**	2.0 [2.0–2.0]	2.0 [2.0–2.0]	2.0 [2.0–2.0]	0.890
**Gender-related barriers to care, median [IQR]**	0.0 [0.0–0.3]	0.0 [0.0–0.3]	0.0 [0.0–0.3]	0.266
**Attitude toward returning to the clinic for CD4+ count results, median [IQR]**	5.0 [4.0–5.0]	4.5 [4.0–5.0]	5.0 [4.0–5.0]	0.002

^1^ p-value for chi-squared statistic or Mann-Whitney U test, as appropriate

Overall, 126 (27.5%) individuals had their blood drawn for CD4+ count on the same day as receiving their HIV+ test result and 333 (72.5%) did not (Table 1). Those who had a day-of-diagnosis blood draw were significantly older, significantly more likely to be working and to have an income source, lived significantly farther away from the clinic, and had a significantly less positive attitude about returning to the clinic to obtain CD4+ count results.

Of those who did not have a day-of-diagnosis blood draw for CD4+ count, clinic-related reasons for not having it were cited by 48.3% of the 242 who replied to this question (Table 2). These included ‘clinic or lab was closing’ (20.7%) and ‘told by staff to return another day’ (14.0%). Personal reasons were cited by 51.7%, including ‘too shocked or upset, afraid to learn CD4+ results’ (9.1%), ‘family or work obligations’ (9.5%), and ‘did not have time to stay’ (18.6%). No participant cited more than one reason.

**Table 2:** Reasons for no CD4+ count blood draw on days of diagnosis among newly-diagnosed HIV+ women and men, Durban South Africa, 2010–2012.

	N	%	Category %
**Clinic-related Reasons**			
Clinic or lab too full	8	3.3%	
Too late—Clinic or lab closed/closing	50	20.7%	
Told by staff to return another day	34	14.0%	
Unsure of what to do next or not told to get CD4+ count	18	7.4%	
Did not have correct clinic documents	7	2.9%	48.3%
**Personal Reasons**			
Too shocked or upset, or afraid to learn CD4+ results	22	9.1%	
Prefer CD4+ testing at a different clinic	5	2.1%	
Prefer to return another day	18	7.4%	
Family or work obligations (no one to care for people at home, unable to take time from work)	23	9.5%	
Did not have time to stay	45	18.6%	
Other (felt too ill, wanted someone to accompany them, personal problem)	12	5.0%	51.7%
**Total**	242	100.0%	

**Note:** 89 of the 333 who did not have blood drawn at diagnosis were not asked this question, as their blood draw had been completed by the time of their baseline interview, and 2 responses were missing.

### Time to linkage to care by blood draw for CD4+ count at diagnosis

**Fig 2:**
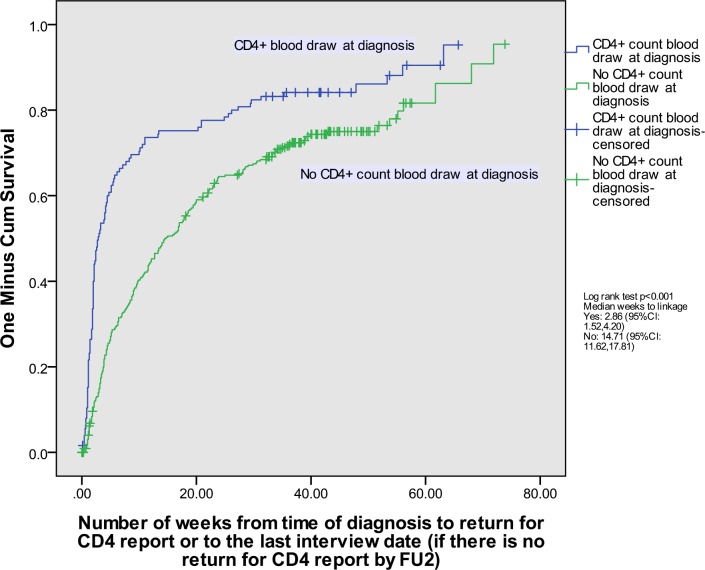
Time to linkage to care by CD4+ count blood draw at diagnosis, newly-diagnosed HIV+ South African women and men.

**Fig 3:**
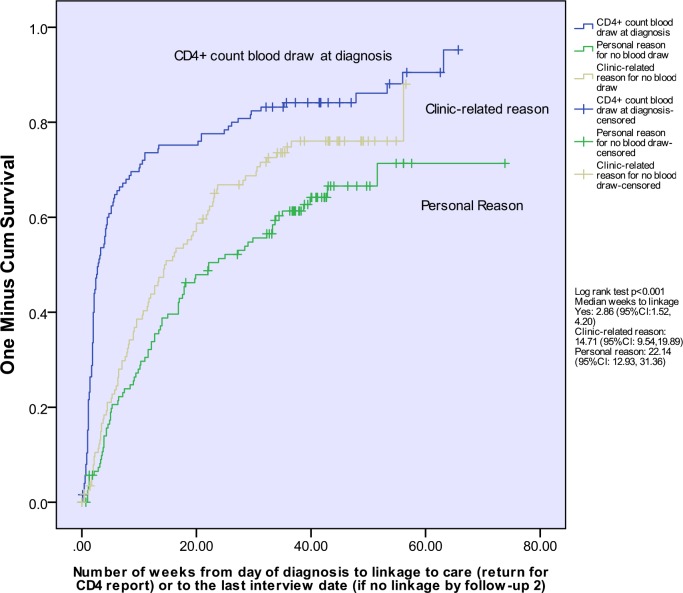
Time to linkage to care by CD4+ count blood draw at diagnosis and reason for no blood draw, newly-diagnosed HIV+ South African women and men.

Of those who did not have their blood drawn on the day of diagnosis, 19.2% reported never returning for this test and, therefore were lost to care. The Kaplan Meier curves for time to linkage to care for those without and with a day-of-diagnosis blood draw are shown in Fig 2. The median time to linkage was 14.71 weeks (95% confidence interval [CI]: 11.62, 17.81) for those without a day-of-diagnosis CD4+ count blood draw and 2.86 weeks (95%CI: 1.52–4.20) for those with this test (log-rank test *p*< 0.001). Kaplan-Meier curves for all three groups—those who had a day-of-diagnosis blood draw, and those who did not because of clinic-related or personal reasons are shown in Fig 3. For those with a clinic-related reason the median time to linkage was 14.71 weeks (95%CI: 9.54–19.89), and for those with a personal reason the median time to linkage was 22.14 weeks (95%CI: 12.93, 31.36) (log-rank test *p* <0.001).

In Cox proportional hazards models, the crude hazard ratio for linkage (HR_linkage_) associated with not having a day-of-diagnosis CD4+ count was 0.60 (95%CI: 0.47, 0.78) (Table 3, upper left panel). The adjusted HR (AHR_linkage_) including confounding variables (age, distance from home to clinic by roads, participant suspected being HIV+, and positive attitude toward returning to the clinic to collect test results) was 0.66 (95%CI: 0.51, 0.85), indicating that the hazard rate of linkage to care of those without a day-of-diagnosis blood draw was 34% lower than that of those who had this test on their day-of-diagnosis.

**Table 3:** Linkage to care by day-of-diagnosis blood draw for CD4+ count and reasons for no day-of-diagnosis blood draw, newly-diagnosed HIV+ women and men, Durban, South Africa, 2010–2012.

		Hazard Ratio of Linkage^1^		Percent Linked to Care				Odds Ratio of Linkage by 4 months^5^		Odds Ratio of Linkage by 8 months^5^	
	N (%)	Crude HR^2^ (95% CI) p-value	Adjusted HR^2^^,^^3^ (95% CI) p-value	% Linked to Care by 4 months^4^	M-H x^2^ *p*	% Linked to Care by 8 months^4^	M-H x^2^ *p*	Crude OR^6^ (95% CI) p-value	Adjusted OR^3^^,^^6^ (95% CI)p-value	Crude OR^6^ (95% CI) p-value	Adjusted OR^3^^,^^6^ (95% CI) p-value
**Blood Draw for CD4+ count**											
Blood draw on the day of diagnosis	126 (27.5%)	Reference	Reference	75.2%	0.004	83.2%	0.22	Reference	Reference	Reference	Reference
No blood draw on the day of diagnosis	333 (72.5%)	0.60 (0.47, 0.78) <0.001	0.66 (0.51, 0.85) 0.001	54.8%		71.7%		0.46 (0.28, 0.76) 0.003	0.52 (0.32, 0.87)0.013	0.63 (0.35, 1.12)0.117	0.76 (0.42, 1.37)0.352
		**Hazard Ratio of linkage**^**1**^		**Percent Linked to Care**				**Odds Ratio of Linkage by 4 months**^**5**^		**Odds Ratio of Linkage by 8 months**^**5**^	
**Reason for no CD4+ blood draw on the day of diagnosis**	**N**^**7**^ **(%)**	**Crude HR**^**8**^ **(95% CI) p-value**	**Adjusted HR**^**3**^^,^^**8**^ **(95% CI) p-value**	**% Linked to Care by 4 months**^**9**^	** **	**% Linked to Care by 8 months**^**9**^	** **	**Crude OR**^**10**^ **(95% CI) p-value**	**Adjusted OR**^**3**^^,^^**10**^ **(95% CI) p-value**	**Crude OR**^**10**^ **(95% CI) p-value**	**Adjusted OR**^**3**^^,^^**10**^ **(95% CI) p-value**
Blood draw on the day of diagnosis	126 (34.2%)	Reference	Reference	75.2%	*	83.2%	*	Reference	Reference	Reference	Reference
Clinic-related reason for no blood draw	117 (31.8%)	0.59(0.43, 0.80) 0.001	0.60(0.44, 0.82) 0.002	55.0%		74.8%		0.45 (0.25, 0.83) 0.01	0.48 (0.26, 0.88) 0.020	0.68 (0.34, 1.36) 0.279	0.76 (0.38, 1.52) 0.434
Personal reason for no blood draw	125 (34.0%)	0.49 (0.35, 0.68) <0.001	0.53(0.38, 0.75) <0.001	44.1%		61.0%		0.34 (0.18, 0.61) <0.001	0.37 (0.20, 0.68) 0.001	0.43 (0.22, 0.83) 0.013	0.51 (0.26, 1.01) 0.054

^1^Estimated from a Cox proportional hazards model.

^2^ N = 453; 6 participants were missing data on distance to clinic.

^3^Adjusted for age in years, distance to clinic, suspected being HIV, attitude for returning to clinic for CD4+ results. Note that besides blood draw at diagnosis, only age was significantly associated with linkage to care in the adjusted models.

^4^ N = 439; 20 participants were missing data on linkage to care

^5^ Estimated from a conditional logistic regression model.

^6^N = 433, 20 participants were missing data on linkage to care and 6 were missing data on distance to clinic.

^7^N = 368, 91 participants were missing data: 89 were not asked this question because they had a blood draw for CD4+ count by the baseline interview, and 2 were missing responses.

^8^ N = 362, 6 participants were missing data on distance to clinic.

^9^ N = 354 participants; 20 participants were missing data on linkage to care.

^10^ N = 348 participants; 20 participants were missing data on linkage to care and 6 were missing data on distance to clinic.

* Mantel-Haenszel chi-squared statistic could not be calculated.

A clinic-related reason for no day-of-diagnosis blood draw was associated with a crude HR_linkage_ of 0.59 (95% CI: 0.43, 0.80) relative to those with a day-of-diagnosis blood draw; a personal reason was associated with a crude HR_linkage_ of 0.49 (95%CI: 0.35, 0.68) (Table 3, lower left panel) The respective AHR_linkage_ for a clinic-related reason was 0.60 (95%CI: 0.44, 0.82), and for a personal reason it was 0.53 (95%CI: 0.38, 0.75), indicating that compared with individuals who had a day-of-diagnosis blood draw, both groups not receiving a day-of-diagnosis blood draw had lower rates of linkage.

The dichotomous outcomes also are shown in Table 3 (center and right columns). By four months after diagnosis, 54.8% of those without a day-of-diagnosis CD4+ count blood draw versus 75.2% of those with one were linked to care (M-H chi-squared *p* = 0.004). By eight months, 71.7% of those without a day-of-diagnosis blood draw vs. 83.2% of those with were linked to care (M-H chi-squared *p* = 0.220). The respective adjusted odds ratio for linkage by four months (AOR_4mo.linkage_) was 0.52 (95%CI:0.32, 0.87) and by eight months, AOR_8mo.linkage_ was 0.76 (95%CI:0.42, 1.37).

Considering reasons for no day-of-diagnosis blood draw, AOR_4mo.linkage_ for a clinic-related reason was 0.48 (95%CI: 0.26, 0.88) and AOR_4mo.linkage_ for a personal reason was 0.37 (95%CI: 0.20, 0.68) relative to those who had this test on the day-of-diagnosis. By eight months, the odds of linkage were lower for both clinic-related and personal reasons but no longer significantly different from those with a day-of-diagnosis blood draw (AOR_8mo.linkage_ for clinic-related reasons = 0.76; 95%CI: 0.38, 1.52; AOR_8mo.linkage_ for personal reasons = 0.51; 95%CI 0.26, 1.01).

In analyses stratified by gender (not shown in table), the AHR_linkage_ for day-of-diagnosis blood draw among women was 0.70 (95%CI: 0.52, 0.95, p = 0.025); among men, it was 0.51 (95%CI: 0.30, 0.86, p = 0.012). Thus, the effect was significant among both groups, and the interaction term was not significant (p = 0.54). The pattern was similar when the predictor was ‘reason for no blood draw’. Compared with day-of-diagnosis blood draw, clinic-related and personal reasons were significantly associated with lower rates of linkage among both women and men [Women: AHR_linkage_ for clinic-related reasons = 0.69 (95%CI: 0.47, 1.01, p = 0.054) and AHR_linkage_ for personal reasons = 0.58 (95%CI: 0.39, 0.86, p = 0.006); Men: AHR_linkage_ for clinic-related reasons = 0.40 (95% CI: 0.21, 0.75, p = 0.005) and AHR_linkage_ for personal reasons = 0.25 (95% CI: 0.11, 0.58, p = .001).] The p-value associated with the interaction term was 0.40.

## Discussion

In this prospective study that identified individuals prior to HCT and followed them for eight months from the day of diagnosis with high retention rates, we found that newly-diagnosed HIV+ individuals who did not have their blood drawn for CD4+ count on the same day as they were diagnosed had a significantly lower rate of linkage to care than those who did, and that both clinic-related and personal reasons for not having this test on the day of diagnosis were associated with a significantly lower rate of linkage. The effects were similar for women and men. Moreover, almost one-fifth of those who did not have this test done on their day of diagnosis never returned.

There are three important implications of these findings. First, this study highlights the importance of identifying very early losses to care. Regardless of the reason, leaving the clinic without completing all the recommended services for that visit seemed to be a marker for being at risk for delayed or no return. Tracking HIV+ individuals enrolled in care—both pre-ART and after initiating ART—for potential loss to follow-up is now an accepted best practice [23], and our study demonstrates that this practice needs to be conducted from the point of diagnosis, even if ART is initiated early in care.

Second, clinic-related barriers were important in explaining about half of the cases with no day-of-diagnosis blood draw. Clinic procedures can make a difference in individuals’ ability to complete necessary services, especially in settings where poverty-related barriers may make it difficult to attend the clinic. Once identified, clinic-related barriers often can be addressed through changes in procedures.

In this study 35% of individuals who did not have blood for CD4+ count drawn at diagnosis indicated that the clinic or lab was closing, or that they were told by staff to return another day. When we presented study findings to clinic staff, they explained that CD4+ blood draws were sometimes deferred because the van collecting specimens for transport to the laboratory had already left on that day, and blood was not stored in the clinic overnight. However, new evidence suggests this barrier could be eliminated without great expense; Skhosana et al. demonstrated high reproducibility of CD4+ count results of blood refrigerated overnight [24]. If those findings are replicated, arriving ‘late in the day’ would no longer be a barrier for individuals to have an immediate blood draw for CD4+ count.

Third, personal reasons for leaving the clinic before having blood drawn for CD4+ count also were important, and these require further investigation, both in research and in practice. Although some explanations lacked detail, they likely reflect psychological processes related to receiving a diagnosis of a life-threatening and highly stigmatized disease, along with structural barriers related to work and family responsibilities. Such barriers may continue to exert an influence even if disease-staging prior to initiation ART is eliminated. Therefore, our findings also highlight how vital it is to ensure that newly-diagnosed HIV+ individual leave the clinic with a basic package of linkage services–immediate psychological support, an assessment of barriers to returning to the clinic, and a plan for follow-up contact and support. These services often are not routine in busy public sector clinics such as those where we conducted this study, but are essential to successful linkage to care.

The findings of this study also are notable for their consistency across women and men. Whereas other research has shown that men are at greater risk than women for late diagnosis and loss to care [5,20], the effects of having a day-of-diagnosis blood draw (and of not doing so for clinic-related and personal reasons) did not differ significantly between women and men.

Strengths of this study include that it was a prospective cohort study representative of people undergoing HCT at these clinics, with close to a 60% of those eligible joining the study. Minimal differences between eligible participants who joined and did not join—by clinic and relationship status—were unlikely to have altered these findings, as all analyses were stratified by clinic, and relationship status was not associated with the exposure or outcome. Additional strengths included that we identified potential study participants and assessed potentially confounding variables prior to diagnosis, had less than 4% loss to follow-up, and that we obtained detailed information about dates, locations, and reasons for all clinic visits during the follow-up period. A limitation was that we relied on participant self-report of clinic visits and tests, including both the exposure and outcome. In these public primary care clinics individual medical records were not created until an HIV+ person initiated ART. Although dates of blood draw could have been obtained from the National HIV laboratory system, we did not use this source because it lacked data on our primary outcome–return to the clinic to obtain CD4+ count test results. Due to the careful way that we elicited visit data, we believe they were comparable in completeness and accuracy to what we would have obtained through clinical records had they existed. However, having multiple sources from which to obtain and validate this information would have increased accuracy.

Other limitations are that we did not probe individuals’ explanations for why they did not have a day-of-diagnosis blood drawn for CD4+ count and we did not ask this question of people who had had this test done by the time of their baseline interview. Additionally, although we did not provide clinic visit reminders, payments to individuals when they returned for study interviews (which were conducted close to clinics of recruitment) may have encouraged them to keep clinic visits. Our estimates of time to linkage, therefore, may be higher than what would have occurred without the study.

In summary, in a detailed analysis made possible by interviewing individuals beginning when they first presented for HCT, we have shown that adherence to the South African health service guideline for immediate blood draw for CD4+ count on the day of diagnosis was less than optimal in these clinics, and was associated with substantial loss to follow-up and delayed linkage to care. Engaging clinic staff in identifying and removing clinic-related barriers to these first steps in care can be an important tool for achieving the 90-90-90 targets set forth by UNAIDS [25]. Further exploration of the personal reasons that newly-diagnosed individuals reported for leaving the place of diagnosis without completing all procedures could help identify better approaches for providing support at this critical stage.

## Supporting Information

S1 Data Underlying Data. (XLSX)

S1 Table Description of the population at screening, overall and by enrollment status, Durban, South Africa, 2010–2012. (DOCX)
