# Supplementary material for: Immediate Blood Draw for CD4+ Cell Count Is Associated with Linkage to Care in Durban, South Africa: Findings from Pathways to Engagement in HIV Care
Source: PLoS One. 2016 Oct 5;11(10):e0162085. doi: 10.1371/journal.pone.0162085 (PMC5051894; doi:10.1371/journal.pone.0162085)
Supplement: S1 Table — (DOCX) [file pone.0162085.s002.docx]

| **S1 Table. Description of the population at screening, overall and by enrollment status, Durban, South Africa, 2010-2012.** | | | | |
| --- | --- | --- | --- | --- |
|  |  |  |  |  |
|  | **Eligible and did not enroll** | **Enrolled in cohort study** | **p-value^1^** |  |
|  | **N = 326** | **N = 459** |  |  |
| **DEMOGRAPHIC AND SOCIOECONOMIC CHARACTERISTICS** |  |  |  |  |
| **Gender, %** |  |  |  |  |
| Women | 66.9 | 67.3 | 0.956 |  |
| Men | 33.1 | 32.7 |  |  |
| **Age in years, median [IQR]** | 28 [24-34] | 29 [25-35] | 0.183 |  |
| **Agegroup, %** |  |  |  |  |
| <=24 | 25.8 | 21.6 | 0.171 |  |
| 25 to 28 | 24.5 | 21.4 |  |  |
| 29 to 31 | 14.1 | 18.1 |  |  |
| 32 to 36 | 14.7 | 19.2 |  |  |
| >=37 | 20.9 | 19.8 |  |  |
| **Relationship status,%** |  |  |  |  |
| Not married/in a relationship | 10.7 | 11.1 | 0.032 |  |
| Married/in a relationship - not living together | 56.4 | 64.5 |  |  |
| Married/in a relationship - living together | 32.8 | 24.4 |  |  |
| **Number of children living with you, median [IQR]** | 1 [0-2] | 1 [0-2] | 0.339 |  |
| **Highest year of school,%** |  |  |  |  |
| 8th grade or less | 27.0 | 25.1 | 0.245 |  |
| 9th-11th grade | 42.9 | 48.6 |  |  |
| Matriculated (completed h.s.) | 20.9 | 16.1 |  |  |
| More than high school | 9.2 | 10.2 |  |  |
| **Employment,%** |  |  |  |  |
| Employed full/part time or self-employed | 34.8 | 30.5 | 0.237 |  |
| Unemployed, unable to work, student | 65.2 | 69.5 |  |  |
| **Food insecurity, %** |  |  |  |  |
| Never | 59.5 | 53.6 | 0.261 |  |
| Seldom | 10.7 | 10.5 |  |  |
| Sometimes | 29.6 | 23.1 |  |  |
| Often | 9.2 | 12.9 |  |  |
| **Income Source,%** |  |  |  |  |
| Has income source or government grant | 96.3 | 96.3 | 1.000 |  |
| No income source & no government grant | 3.7 | 3.7 |  |  |
| **Travel time to clinic, %** |  |  |  |  |
| <1/2 hour | 51.9 | 45.5 | 0.095 |  |
| ≥1/2 hour | 48.1 | 54.5 |  |  |
| **TESTING CHARACTERISTICS** |  |  |  |  |
| **Ever tested (negative) before this test, %** |  |  |  |  |
| Yes | 35.7 | 38.6 | 0.443 |  |
| No | 64.3 | 61.4 |  |  |
| **Suspected HIV-positive, %** |  |  |  |  |
| Yes/unsure | 54.6 | 61.0 | 0.086 |  |
| No | 45.4 | 39.0 |  |  |
| **Number of HIV-related symptoms, %** |  |  |  |  |
| None | 56.4 | 53.2 | 0.636 |  |
| One | 18.4 | 20.5 |  |  |
| More than one | 25.2 | 26.4 |  |  |
| **PSYCHOSOCIAL and COGNITIVE FACTORS** |  |  |  |  |
| **Symptoms of psychological distress, %** |  |  |  |  |
| Not elevated (<16) | 88.3 | 87.7 | 0.894 |  |
| Elevated (> 16) | 11.7 | 12.3 |  |  |
| **HIV-related stigma, median [IQR]** | 2 [2.0-2.0] | 2.0 [2.0-2.0] | 0.266 |  |
| **Gender-related barriers to care, median [IQR]** | 0 [0.0-0.3] | 0 [0.0-0.3] | 0.593 |  |
| **Attitude toward returning to the clinic for CD4+ count results, median [IQR]** | 5.0 [4.0-5.0] | 5.0 [4.0-5.0] | 0.287 |  |
|  | | |  |  |

**^1^ p-value for chi-squared statistic or Mann-Whitney U test, as appropriate**
